# Physical activity and mental health in children and adolescents during and after the COVID-19 pandemic: findings from the six-wave German COPSY study

**DOI:** 10.3389/fspor.2026.1776510

**Published:** 2026-04-08

**Authors:** Steven Behn, Ann-Kathrin Napp, Franziska Reiß, Anne Kaman, Claus Barkmann, Michael Erhart, Ulrike Ravens-Sieberer

**Affiliations:** 1 Department of Child and Adolescent Psychiatry, Psychotherapy, and Psychosomatics, University Medical Center Hamburg-Eppendorf, Hamburg, Germany; 2 Alice Salomon University of Applied Science, Berlin, Germany

**Keywords:** children and adolescents, COVID-19 pandemic, health-related quality of life, mental health problems, physical activity, well-being, youth

## Abstract

**Background:**

The COVID-19 pandemic coincided with declines in youth mental health and health-related quality of life. Physical activity (PA) has been identified as a modifiable factor associated with better mental health outcomes, yet longitudinal evidence jointly examining well-being and mental health problems remains limited.

**Methods:**

Data stem from six waves of the German COPSY cohort of 11- to 21-year-olds (*N* = 1,819) surveyed during and after the COVID-19 pandemic. PA (self-reported days/week ≥60 min) was categorized as low (0–2 days), medium (3–5), high (6–7). Outcomes were health-related quality of life (KIDSCREEN-10, parent-report) and mental health problems (SDQ, parent-report). Linear mixed models with random intercepts and slopes included PA, time, age, gender, socioeconomic status, and current mental disorder. Two-way interactions between PA and covariates were tested, and sensitivity analyses used a WHO guideline indicator (≥60 min/day).

**Results:**

Higher PA was dose-responsively associated with better health-related quality of life and fewer mental health problems across all waves. Compared to low PA, higher PA levels related to small to moderate improvements in health-related quality of life and small reductions in mental health problems. A small interaction indicated that the association between high PA and fewer mental health problems was slightly stronger among older adolescents; all other interactions were non-significant. Sensitivity analyses yielded comparable results.

**Conclusions:**

PA showed small to moderate associations with higher health-related quality of life and fewer mental health problems during and after the pandemic. Ensuring accessible opportunities for youth PA should be integral to mental health promotion and future crisis preparedness.

## 1 Introduction

The COVID-19 pandemic profoundly disrupted the daily lives of children and adolescents worldwide, leading to social isolation, school closures, and increased family stress. International reviews have documented substantial declines in health-related quality of life (HrQoL) and increases in mental health problems during the early pandemic period (1, 2). Longitudinal and repeated cross-sectional studies suggest that these difficulties peaked during periods of lockdowns and school closures (3–5).

In Germany, findings from the longitudinal COVID-19 and Psychological Health (COPSY) study mirror these global patterns: the proportion of children and adolescents with low HrQoL rose from 18% pre-pandemic to 48% during the pandemic (6, 7). Likewise, up to 31% of children and adolescents experienced mental health problems during the beginning of the pandemic, compared to 18% before the pandemic. These burdens were particularly pronounced among socially disadvantaged children and adolescents (8), who were already at elevated risk for poor mental health prior the pandemic (9). Beyond COVID-19, other global crises—including wars, climate change, and economic instability—have increasingly become concerns for young people and may further threaten their mental health and well-being (10). Identifying modifiable protective factors that support psychological well-being and mental health under such conditions is therefore an urgent public health priority.

Physical activity (PA) has emerged as a promising coping resource associated with better mental health outcomes in times of crisis. PA is defined as any bodily movement produced by skeletal muscles that requires energy expenditure and encompasses sport, recreational play, active transport, and household tasks (11). The 2010 WHO guidelines recommend that children and adolescents aged 5–17 years engage in at least 60 min of moderate-to-vigorous PA daily, including muscle- and bone-strengthening activities several times per week (12). Even before the pandemic, however, global surveillance based on self-report indicated that fewer than 20% of 11–17-year-olds met these recommendations (13), while accelerometer-based monitoring in Europe suggested that around two-thirds of 10- to 18-year-olds did not reach recommended PA levels (14). These divergent estimates illustrate that prevalence estimates are highly dependent on the measurement method used; self-report is generally considered less precise than device-based assessment and tends to overestimate guideline adherence in youth (15, 16). While self-report remains the most feasible approach in large population-based surveys, findings on guideline adherence should be interpreted accordingly. Pandemic-related restrictions, including school closures and the suspension of organized sports, further exacerbated PA declines (17, 18). This concern is also highlighted by recent German data from the Health Behaviour in School aged Children (HBSC) survey: only 11% of girls and 21% of boys aged 11–15 met WHO recommendations, while half of adolescents were classified as having low PA (0–2 days/week) (19). Taken together, these findings highlight a persistent and growing public health concern: despite well-established physical and psychological benefits of PA for youth development (20), most young people do not meet recommended PA levels.

The mental health benefits of PA are well-supported by current evidence. Meta-analyses and systematic reviews consistently link higher PA with fewer depressive and anxiety symptoms and better emotional and behavioral functioning (21–23). During the COVID-19 pandemic, several reviews similarly reported that more active children and adolescents exhibited fewer depressive and anxiety symptoms and higher well-being (24–28). Lubans et al. (29) conceptual framework outlines neurobiological (e.g., enhanced brain function), behavioral (e.g., improved sleep), and psychosocial (e.g., self-esteem, social connectedness) pathways through which PA may influence mental health. Importantly, the model emphasizes the dual role of PA in promoting well-being and reducing ill-being—underscoring the importance of examining a broad range of mental health outcomes.

While much of the existing literature on PA and youth mental health has examined internalizing outcomes such as anxiety and depression, broader indicators such as behavioral problems and HrQoL have received growing but still comparatively less attention (20). Recent reviews have identified consistent associations between higher PA and both fewer general mental health problems (30) and higher HrQoL (31, 32). However, most studies assess either well-being or mental health problems in isolation, and integrated longitudinal assessments of both domains within the same study remain scarce. Notable exceptions include longitudinal evidence from Australia showing that increases in PA predicted subsequent gains in HrQoL and reductions in socio-emotional difficulties (33), and a German school-based study that concurrently assessed HrQoL, mental health problems, and device-based PA during pandemic recovery (34). Building on these foundations, further research is needed to clarify the extent to which PA is linked to both positive and negative dimensions of youth mental health across multiple pandemic and post-pandemic phases.

Individual characteristics such as age, gender, and socioeconomic status (SES) may shape both PA participation and mental health outcomes by influencing access to resources, exposure to stressors, and coping strategies (13, 35). Because SES is associated with both PA levels and mental health outcomes (9, 36), it represents a potential confounding variable. A key analytical question is therefore whether PA explains variance in mental health outcomes beyond what can already be attributed to sociodemographic differences. Lubans et al.´s (29) framework also suggests that the relationship between PA and mental health outcomes may vary across individual and contextual factors (29). Girls and older adolescents are less likely to meet PA guidelines, and adolescents from lower-SES families tend to report lower HrQoL and more mental health problems (9, 37, 38). Pandemic phases further differed in their public health restrictions and opportunities for PA, potentially altering the strength of PA-mental health associations over time. It therefore remains unclear whether PA's mental health benefits during crises are uniform across subgroups or phases, or whether they are amplified among groups with lower baseline PA and higher psychosocial vulnerability, such as girls, older adolescents, or those from lower-SES backgrounds. By including age, gender and SES as covariates alongside PA in our multivariate models, we can estimate the unique contribution of PA to mental health outcomes after accounting for these sociodemographic differences.

Against this background, the present study examines both HrQoL and general mental health problems to capture positive and negative dimensions of youth mental health, while accounting for key individual characteristics (age, gender, SES) and clinical status (current mental disorder) that may confound or moderate these associations. Our population-based study of German children and adolescents extends previous work by jointly modelling PA with both well-being and ill-being across multiple pandemic and post-pandemic phases. From a public-health perspective, this comprehensive approach is essential: well-being and ill-being are related yet distinct constructs, and PA promotion efforts should therefore be evaluated by their potential both to reduce symptom burden and to strengthen positive functioning. By clarifying the role of PA as a modifiable coping resource during crises, this study aims to inform public health strategies for promoting mental health, resilience, and well-being among youth.

Despite growing evidence on the benefits of PA for youth mental health, longitudinal studies that ex-amine its role in relation to both positive and negative mental health indicators across multiple pan-demic phases remain scarce. This study addresses this gap by investigating the following research questions:

Research question 1: How did trajectories of HrQoL and mental health problems during and after the COVID-19 pandemic differ across PA groups (low, medium, high)?

H1a: Children and adolescents with higher PA levels (medium, high) show consistently higher HrQoL over time than those with low PA.
H1b: Children and adolescents with higher PA levels (medium, high) show consistently fewer mental health problems over time than those with low PA.

Research question 2: To what extent are different levels of PA associated with HrQoL and mental health problems over time in multivariable models, and do these associations differ by age, gender, SES, and pandemic phase?

H2a: Higher PA levels are associated with higher HrQoL and fewer mental health problems over time, even after adjustment for age, gender, SES, and current mental disorder.
H2b: The strength of the association between PA and mental health outcomes differs across subgroups and pandemic phases, with stronger associations expected for girls, older adolescents, those from lower SES backgrounds, and during phases of stricter public health restriction.

## 2 Materials and methods

### 2.1 Study design and sample

The present study draws on six waves of the German population-based COPSY study, which investigates the mental health and well-being of children and adolescents during and after the COVID-19 pandemic. Data were collected at T1 (May–June 2020; partial lockdowns including school closures and suspension of organized sports and leisure activities), T2 (Dec 2020–Jan 2021; nationwide full lockdown with schools and sports clubs closed), T3 (Sept–Oct 2021; after a summer with lower infection rates), T4 (Feb 2022; second pandemic winter and after the onset of the war in Ukraine), T5 (Sep–Oct 2022; period of high energy prices and inflation), T6 (Oct–Nov 2023; after the pandemic was officially declared over).

**Figure 1:**
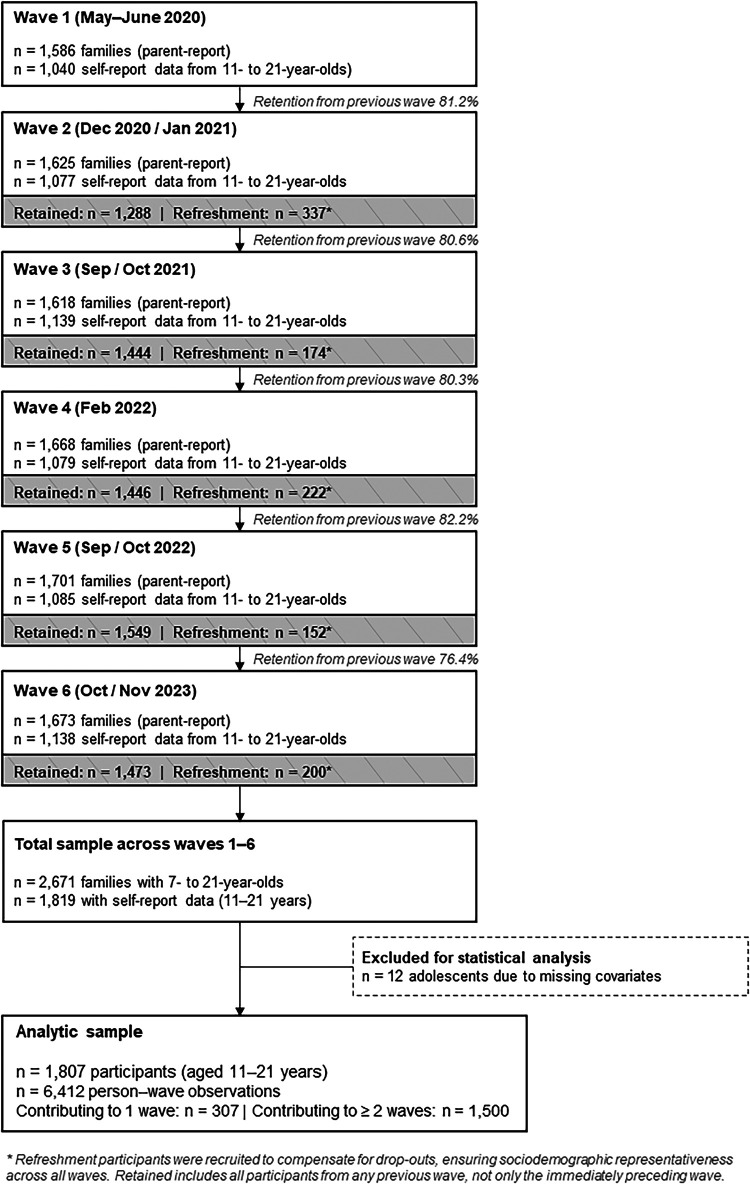
Participant flow diagram.

Figure 1 depicts the participant flow across all six study waves, including sample composition, retention rates, and exclusions for the analytic sample. Across these waves, between *n* = 1,040 (T1) and *n* = 1,137 (T6) children and adolescents aged 11–21 years as well as *n* = 1,586 (T1) and *n* = 1,673 (T6) parents participated in online surveys. Families were invited via email through a large online panel using quota sampling to ensure that the sample reflected the sociodemographic characteristics of the German population. Participants who had previously taken part were re-invited for subsequent waves, and additional families were recruited to maintain representativeness. In total, *n* = 2,671 families participated in at least one wave, with self-reported data available from *n* = 1,819 adolescents (11–21 years). Analyses included all person–wave observations with concurrent self-reported PA and the respective parent-reported outcomes. The study design is based on the German BELLA study (39), the mental health module of the German National Health Interview and Examination Survey among Children and Adolescents (KiGGS). Further methodological details are reported in previous publications of the project (6, 40).

Retention rates among participants with continuous participation were high, ranging from 76% to 82% across waves (Figure 1). To assess potential selective attrition, baseline characteristics of participants with higher study participation (≥4 of 6 waves with data; *n* = 1,133) were compared with those with lower participation (<4 waves; *n* = 453) among all T1 participants (*n* = 1,586). Completers were younger (1.2 years) and slightly more likely to have low parental education; both variables are controlled for in all models. For gender, current mental disorder, and PA group distribution, no statistically significant differences were observed. Regarding the mental health outcomes, no significant differences were found for either HrQoL or mental health problems, suggesting that selective attrition did not systematically bias the key outcome variables.

### 2.2 Variables and instruments

**Physical activity.** Self-reported PA was assessed with a validated item from the German MoMo Physical Activity Questionnaire (41, 42), which is part of the representative German KiGGS study. The item operationalizes the WHO recommendation of ≥60 min of moderate-to-vigorous PA for children and adolescents, noting that the updated WHO guidelines express this target as an average of 60 min per day (43). Participants were asked: “*On how many days of a normal week are you physically active for at least 60 min per day (e.g., running, fast walking, cycling, swimming, football)?”*. Responses were categorized into three groups in line with recommendations of the HBSC study (44): low (0–2 days/week), medium (3–5 days/week), and high (6–7 days/week).

**Health-related quality of life.** HrQoL was assessed using the parent-reported KIDSCREEN-10 Index. Items are rated on a five-point response-scale ranging from *not at all* to *extremely*. Rasch modeling yields a unidimensional person score that is transformed into standardized T-scores (*M* = 50, *SD* = 10) based on European KIDSCREEN data to facilitate interpretation (45). The continuous T-Score was used as an indicator of overall HrQoL, with higher scores indicating better HrQoL (46). For descriptive purposes, HrQoL categories (low, medium, high) were derived based on German normative data from the BELLA study (46). The KIDSCREEN-10 is an internationally used questionnaire with good reliability (e.g., Cronbach's *α* = 0.82) and validity (47).

**Mental health problems.** Mental health problems were assessed using the parent-reported Strengths and Difficulties Questionnaire (SDQ; 48). The total difficulty score (20 items across four subscales: emotional symptoms, conduct problems, hyperactivity-inattention, peer problems) was used as the primary continuous outcome in the mixed-effects models (higher scores indicate more problems). Supplementary analyses examined each subscale separately. For descriptive purposes, the distribution across three categories (normal/borderline/abnormal) is reported using established cut-offs (49).

**Covariates.** Covariates were selected *a priori* based on established empirical evidence identifying age, gender, and SES as key confounders of both PA participation and mental health outcomes in youth (9, 13), and the theoretical framework by Lubans et al. (29). They included age (centered at the sample mean), gender (female/male, a small diverse category was excluded for regression analyses due to low cell size), pandemic timing and socioeconomic status (SES). SES was indexed by parental education using the Comparative Analysis of Social Mobility in Industrial Nations (CASMIN) classification (50). The CASMIN classification categorizes educational attainment into three hierarchical levels based on completed educational qualifications: low (inadequately completed general education, general elementary education, or basic vocational training), medium (intermediate education with or without vocational training), and high (completed tertiary education, including university degree). Parental education was used as a proxy for SES, which is a common approach in child and adolescent health research (9), though it captures only one dimension of socioeconomic position. A current diagnosed mental disorder (*yes/no*) was parent-reported. To represent pandemic timing and accommodate varying intervals between waves, a numeric time variable was coded as years since T1 (T1 = 0.00; T2 = 0.50; T3 = 1.33; T4 = 1.67; T5 = 2.33; T6 = 3.42).

### 2.3 Statistical analyses

All analyses were performed in R (version 4.5.0). First, descriptive trajectories of HrQoL (KIDSCREEN-10T-scores) and mental health problems (SDQ total difficulty score) were plotted across the six waves, stratified by PA group (low, medium, high).

To examine longitudinal associations of PA with HrQoL and mental health problems, linear mixed-effects models were estimated using the *lme4* package (51). Each model included a random intercept for each participant to account for within-person clustering. A random slope for the linear time term was tested and retained if model fit improved (likelihood ratio test, AIC/BIC), allowing individual trajectories to vary over time. Time was modelled in the fixed effects using a natural cubic spline with three degrees of freedom to capture non-linear trends and accommodate uneven spacing between waves. Three degrees of freedom were selected based on model comparison; models with two, three, and four degrees of freedom were compared using AIC and BIC. Both criteria converged on three degrees of freedom as the most parsimonious specification. To visualize adjusted group trajectories, estimated marginal means were computed from the final models at each wave.

Fixed effects included PA group, spline-transformed time, grand mean centered age, gender, SES, and current mental disorder. Main effects of PA were estimated to test whether higher PA levels were associated with better HrQoL and fewer mental health problems over time. All hypothesized two-way interactions between PA and the covariates (time, age, gender, SES) were included to examine potential moderation effects. Standardized mean differences (Cohen's d) for pairwise PA contrasts (medium vs. low, high vs. low) are reported to facilitate interpretability. Additionally, a *post-hoc* comparison between the high and medium PA groups was obtained by re-estimating the models with medium PA as the reference category. Deviating from the original Cohen's d formula, the present values were computed as the estimated marginal mean difference divided by the overall standard deviation of the outcome across all person–wave observations. Confidence intervals are presented for unstandardized contrasts. The same model specification was applied to each SDQ subscale (emotional symptoms, conduct problems, hyperactivity-inattention, peer problems) in exploratory analyses to examine whether PA associations differed across symptom domains.

Model building followed a three-step procedure: (1) an unconditional null model was estimated to calculate the intraclass correlation coefficient (ICC) and confirm the need for mixed modeling; (2) a full model including all hypothesized main effects and interaction terms was specified; and (3) non-significant interactions were removed stepwise based on likelihood-ratio tests and fit indices (AIC, BIC, -2LL) to obtain a parsimonious final model.

Missing data arose from wave non-participation due to the open cohort design; there was no item-level missingness among self-reported PA and parent-reported outcomes. Fifteen participants (0.8%) were excluded due to missing covariates. Models were estimated using maximum likelihood, which includes all available person–wave observations and provides unbiased estimates under the Missing at Random assumption. No imputation was applied.

Model assumptions were evaluated through residual diagnostics (normality and heteroscedasticity) and multicollinearity checks. To address minor deviations from normality, 95% confidence intervals were computed via parametric bootstrap (1,000 simulations). Model fit was assessed using AIC, BIC, −2LL, and marginal/conditional R² (*performance* package). Variance inflation factors (VIF) were computed for all predictors in both final models to ensure absence of problematic multicollinearity; all VIFs were below 1.50 (52). Baseline intercorrelations between study variables are reported in the Supplementary Material. The statistical significance was set at *p* < .05.

Sensitivity analyses were conducted to test the robustness of results. These included (a) re-estimating models using a binary indicator for meeting the WHO recommendation (≥60 min/day) instead of PA categories, and (b) obtaining parametric bootstrap confidence intervals (see Supplementary Material) for fixed-effect estimates to verify their stability under potential deviations from normality.

## 3 Results

### 3.1 Sociodemographic characteristics at baseline (T1)

Baseline characteristics are summarized in Table 1. At T1, *N* = 1,040 children and adolescents aged 11–17 years (*M* = 14.3, *SD* = 1.85) participated. Gender was evenly distributed across the sample (51.1% female). Most parents had a medium level of education (56.8%) with 25% reporting high and 18.5% low education. A small minority reported a current mental disorder (6.8%). For HrQoL, nearly half of the sample were classified as having a low (47.6%) or medium (46.8%) HrQoL. According to parent-reports, most of the adolescents were in the normal range of mental health problems, while 29.7% were in the borderline or abnormal range.

**Table 1:** Sociodemographic characteristics of the sample at baseline (wave 1 of the COPSY study).

Variable	*N* (%)
Age^a^	
11–13 years	351 (33.8%)
14–17 years	689 (66.2%)
Gender^a^	
Male	508 (48.8%)
Female	531 (51.1%)
Diverse	1 (0.1%)
Parental education^b^^,^^c^	
Low	288 (18.7%)
Medium	884 (57.3%)
High	383 (24.1%)
Current mental disorder^b^^,^^c^	
Yes	106 (6.7%)
No	1,476 (93.3%)
Physical activity^a^	
Low	586 (56.6%)
Medium	263 (25.3%)
High	191 (18.1%)
Health-related quality of life^b^	
Low	755 (47.6%)
Medium	742 (46.8%)
High	89 (5.6%)
Mental health problems^b^	
Normal	1,115 (70.3%)
Borderline	189 (11.9%)
Abnormal	282 (17.7%)

^a^ Self-report (*N* = 1,040).

^b^ Parent-Report (*N* = 1,586).

^c^ Minor deviations in N due to missing data.

### 3.2 Descriptive trajectories of HrQoL and mental health problems by physical activity group

**Figure 2:**
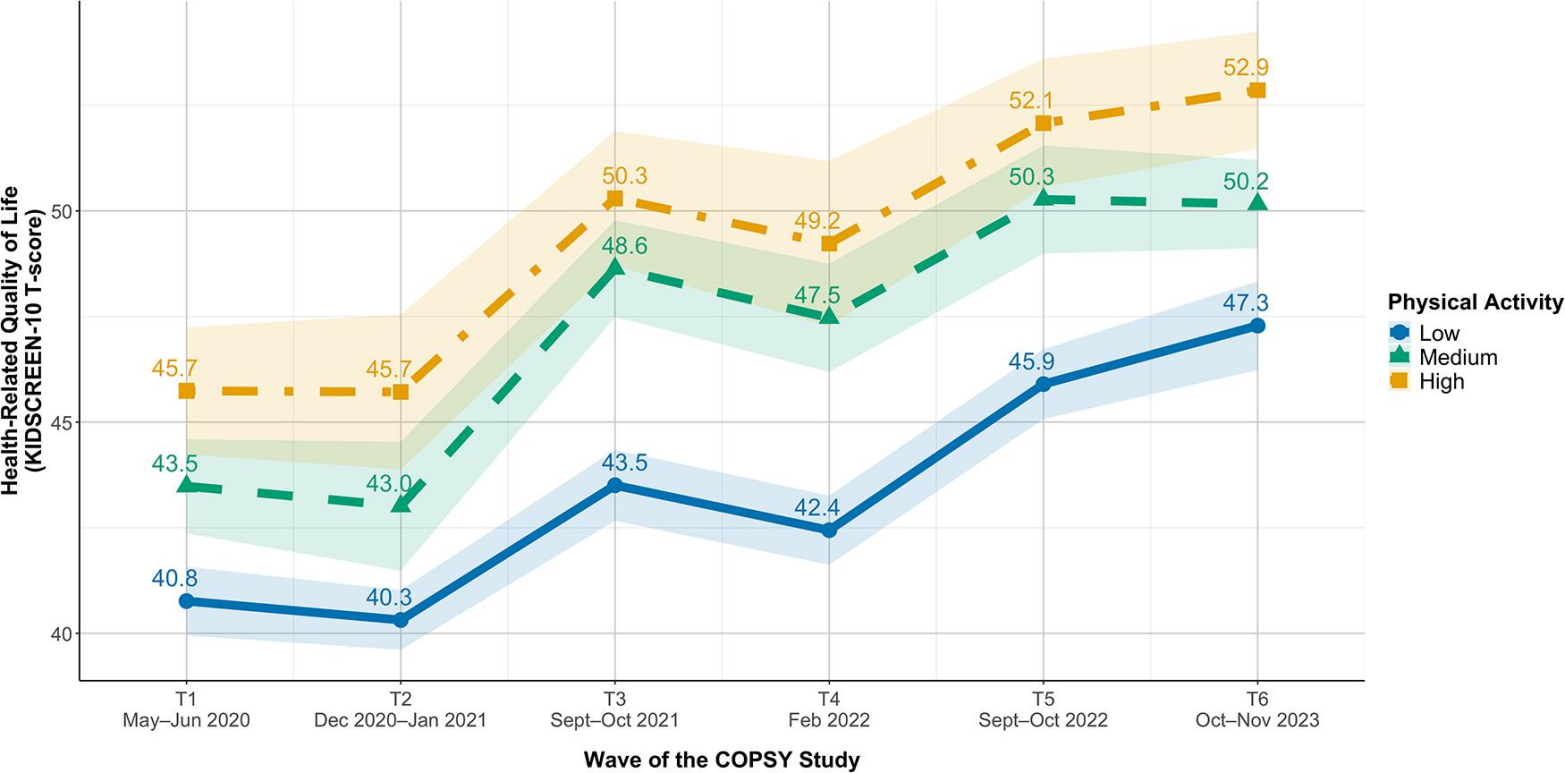
Trajectories of HrQoL in youth in Germany stratified by physical activity group. Values are unadjusted means with 95%–CIs. Higher values indicate better parent-reported HrQoL. Sample sizes varied by wave and PA group.

Figure 2 displays trajectories of HrQoL across the six waves stratified by PA group. All three groups followed a similar non-linear pattern, with the lowest HrQoL during the early restrictive phases (T1, T2) and a marked increase at T3, followed by a slight dip at T4 and continued recovery through T5 and T6. Children and adolescents with high PA consistently showed the highest HrQoL T-scores points (ranging from 45.7 at T1/T2 to 52.9 at T6), followed by the medium (43.0–50.3) and low PA groups (40.3–47.3). The absolute differences between PA groups remained relatively stable across waves, with the gap between low and high PA ranging from approximately 5 to 7 T-score points throughout the study period.

**Figure 3:**
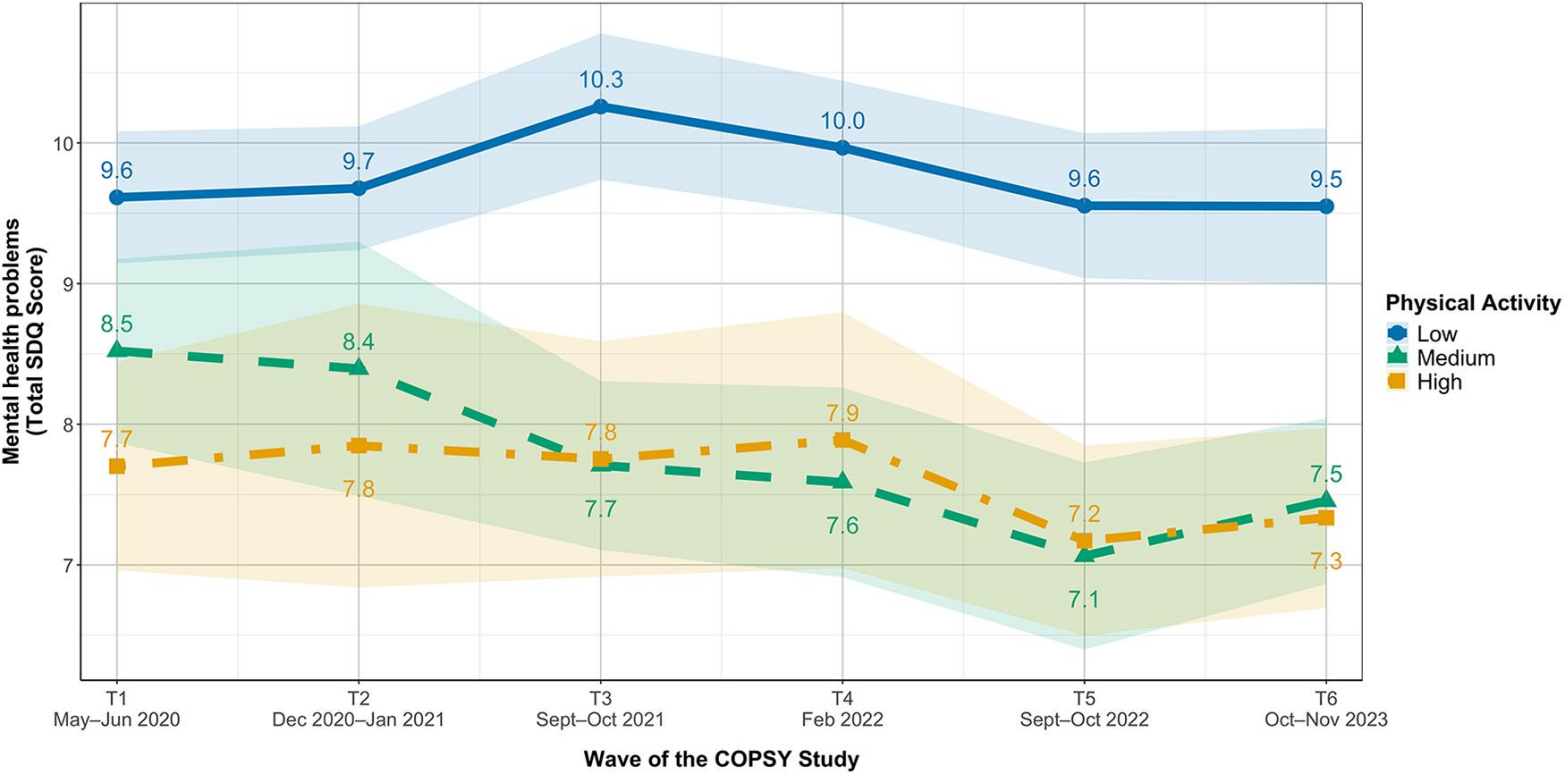
Trajectories of mental health problems in youth in Germany stratified by physical activity group. Values are unadjusted means with 95%–CIs. Higher values indicate higher parent-reported mental health problems. Sample sizes varied by wave and PA group.

Figure 3 shows trajectories of mental health problems by PA group. The medium and high PA groups showed a gradual decline in SDQ scores over time (medium: from 8.5 at T1 to 7.5 at T6; high: from 7.7 to 7.3), whereas the low PA group showed a different pattern, with scores increasing from T1 (9.6) to a peak at T3 (10.3) before returning to baseline levels at T5/T6 (9.5). Consequently, the gap between low and medium/high PA groups widened over time, from approximately 1–2 points at T1 to 2–2.5 points at T5 and T6.

Adjusted trajectories based on estimated marginal means from the final linear mixed models are provided in the Supplementary Material, confirming that group differences persisted after adjustment for age, gender, SES, and current mental disorder.

### 3.3 Linear mixed model results for HrQoL

Table 2 shows the results of the linear mixed model for HrQoL. The unconditional null model yielded an ICC of .54, supporting the use of mixed modeling. The final model with random intercepts, random slopes and natural cubic splines (*df* = 3) for time provided good fit (AIC = 45,781.3; BIC = 45,896.3).

**Table 2:** Results of the linear mixed model for HrQoL in children and adolescents.

Coefficient	*B*	95%—*CI*	*p*-value
Intercept	40.18	(39.25; 41.12)	<.001
Low PA (Ref.)			
Medium PA	2.51	(2.00; 3.02)	<.001
High PA	4.31	(3.70; 4.92)	<.001
Time			
Spline component 1	5.83	(5.05; 6.62)	<.001
Spline component 2	8.87	(7.78; 9.99)	<.001
Spline component 3	6.87	(6.25; 7.49)	<.001
Age (centered)	0.27	(0.09; 0.44)	.003
Male (Ref.)			
Female	0.33	(–0.51; 1.16)	.443
High SES (Ref.)			
Low SES	0.51	(–0.56; 1.59)	.351
Medium SES	–0.32	(–1.12; 0.47)	.427
No current mental disorder (Ref.)			
Current mental disorder	–4.32	(–5.31; –3.33)	<.001
Medium PA × Age	–0.12	(–0.33; 0.08)	.232
High PA × Age	0.20	(–0.04; 0.43)	.099

All coefficients are unstandardized.

PA, physical activity; SES, socioeconomic status.

Time modeled with natural cubic splines (*df* = 3). Models included a random intercept and a random linear time slope (participants as grouping factor), with estimated variances of 64.14 (intercept), 4.07 (slope), and 41.21 (residual). Model fit: *R²_m_* = 0.11; *R²_c_* = 0.65. *N* = 6,412 observations from 1,807 adolescents.

Adolescents who were more physically active consistently showed higher HrQoL. Medium and high PA were associated with small-to-moderate standardized improvements in well-being compared with low PA (*d* = 0.22–0.38). A *post-hoc* comparison between the high and medium PA groups revealed a significant but smaller difference [*b* = 1.80, 95% CI (1.17, 2.44), *p* < .001, *d* = 0.16]. The non-linear time effects captured by the spline terms reflected the overall recovery pattern observed descriptively, with lower HrQoL during more restrictive phases and gradual improvement as restrictions eased. Older age was related to slightly higher HrQoL, while gender and SES showed no significant associations. Adolescents with a current mental disorder had substantially lower HrQoL scores than those without.

All tested interaction terms between PA and moderators (age, gender, SES, time) were non-significant after model selection, including a marginal PA × age effect that did not remain significant in the final model. The explained variance was *R²_m_* = 0.11 and *R²_c_* = 0.65, indicating that both fixed effects and individual differences contributed meaningfully to the overall variance in HrQoL.

### 3.4 Linear mixed model results for mental health problems

Table 3 shows the results of the linear mixed model for mental health problems. The unconditional null model showed an ICC of.72, again supporting a multilevel approach. The final model with random intercepts and random slopes for time captured non-linear changes over time (all spline terms *p* < .001; AIC = 36,715.5; BIC = 36,830.5).

**Table 3:** Results of the linear mixed model for mental health problems in children and adolescents.

Coefficient	*B*	95%—*CI*	*p*-value
Intercept	9.69	(9.18; 10.20)	<.001
Low PA (Ref.)			
Medium PA	−0.73	(–0.98; –0.49)	<.001
High PA	–1.23	(–1.53; –0.94)	<.001
Time			
Spline component 1	–1.14	(–1.51; –0.78)	<.001
Spline component 2	–0.98	(–1.49; –0.47)	<.001
Spline component 3	–1.28	(–1.56; –1.00)	<.001
Age (centered)	–0.41	(–0.50; –0.31)	<.001
Male (Ref.)			
Female	–0.57	(–1.05; –0.09)	.019
High SES (Ref.)			
Low SES	0.67	(0.10; 1.24)	.021
Medium SES	0.37	(–0.04; 0.78)	.080
No current mental disorder (Ref.)			
Current mental disorder	3.52	(3.04; 4.00)	<.001
Medium PA × Age	0.09	(0.00; 0.19)	.058
High PA × Age	0.11	(0.00; 0.23)	.049

All coefficients are unstandardized.

PA, physical activity; SES, socioeconomic status.

Time modeled with natural cubic splines (*df* = 3). Models included a random intercept and a random linea time slope (participants as grouping factor), with estimated variances of 24.56 (intercept), 0.69 (slope), and 9.06 (residual). Model fit: *R²_m_* = 0.06; *R²_c_* = 0.75. *N* = 6,412 observations from 1,807 adolescents.

Higher PA was consistently associated with fewer mental health problems. The associations were small in magnitude but statistically robust, corresponding to standardized effects of approximately *d* = −0.12 for medium and *d* = −0.20 for high PA compared with low PA. The *post-hoc* comparison between high and medium PA was also significant, though smaller in magnitude [b = −0.50, 95% CI (−0.80, −0.20), *p* = .001, *d* = −0.08]. Over time, average SDQ scores declined in a non-linear fashion, suggesting a reduction in symptom burden after pandemic peaks as observed in the descriptive results. Older adolescents and females showed fewer problems, while lower SES was related to slightly higher problem levels. Adolescents with a current mental disorder showed substantially higher SDQ scores than those without.

A small interaction between high PA and age was significant, indicating that the association between high PA and fewer mental health problems was somewhat stronger among older adolescents. All the other tested interactions were non-significant and therefore omitted from the final model. The explained variance was *R²_m_* = 0.06 and *R²_c_* = 0.75, reflecting that most variance stemmed from stable between-person differences, with a smaller but significant share explained by the modeled predictors.

Exploratory analyses of the four SDQ subscales revealed significant PA group differences across all domains (Supplementary Material). Effects were largest for peer problems (medium: *d* = −0.14; high: *d* = −0.19) and emotional symptoms (medium: *d* = −0.10; high: *d* = −0.18), both internalizing domains, and smallest for conduct problems (medium: *d* = −0.09; high: *d* = −0.11) and hyperactivity-inattention (medium: *d* = −0.07; high: *d* = −0.15). A significant PA × age interaction emerged exclusively for peer problems (medium × age: B = 0.040, *p* = .025; high × age: B = 0.053, *p* = .010).

### 3.5 Sensitivity analyses

Results were robust to alternative operationalizations of the self-report PA measure. First, using a WHO guideline indicator (≥60 min/day) instead of PA groups yielded similar patterns: adolescents who met the PA recommendation showed higher HrQoL (*b* = 2.82, *SE* = 0.48, *p* < .001) and fewer mental health problems (*b* = –1.17, *SE* = 0.26, *p* < .001) than those who did not. Second, to address distributional assumptions, parametric bootstraps of fixed effects (1,000 simulations) were conducted in the final models (see Supplementary Material). Bootstrap CIs closely overlapped the asymptotic Wald confidence intervals and did not change statistical inference, supporting the robustness of the estimates under mild deviations from normality.

## 4 Discussion

### 4.1 Summary of findings

In this six-wave, population-based study of German children and adolescents, descriptive trajectories showed that youth with higher levels of PA showed higher HrQoL and fewer mental health problems during and after the COVID-19 pandemic, supporting hypotheses H1a and H1b. Children and adolescents in the high PA group (≥6 days/week) consistently showed higher KIDSCREEN-10 scores and lower SDQ total scores than their low-active peers, with the medium PA group (3–5 days/week) showing similar but smaller differences. These descriptive patterns indicate that physically active youth maintained better psychological adjustment throughout the pandemic phases. In line with H2a, these associations also emerged in fully adjusted mixed-effects models accounting for age, gender, SES, and current mental disorder, suggesting that higher PA was linked to better HrQoL and fewer mental health problems beyond demographic covariates.

### 4.2 Integration with prior evidence

Our findings complement previous studies documenting pandemic-related declines in HrQoL and increases in mental health problems among German youth (1, 2, 7) and extend them by showing that higher PA is linked to better well-being and fewer mental health problems during and after the COVID-19 pandemic. While internalizing outcomes such as anxiety and depressive symptoms have been frequently studied (20, 21, 53), an increasing number of reviews also address broader indicators including well-being, behavioral problems, and quality of life (25, 27, 30). The present study contributes to this broader perspective by demonstrating that PA relates not only to fewer mental health problems but also to higher overall well-being. This aligns with Lubans et al.'s (29) framework which emphasizes the dual role of PA in promoting well-being and reducing ill-being. The observed dose-response pattern—whereby medium and high PA levels yielded progressively more favorable outcomes—supports H1a/b and mirrors recent research showing small but consistent PA-HrQoL associations (54) and robust pandemic-related links between PA and composite mental health (30). Standardized effects in our study were small to moderate for HrQoL and small for mental health problems, indicating that PA was more strongly related to positive well-being than to the reduction of mental health problems. To contextualize the magnitude of the observed associations in practical terms: the KIDSCREEN-10 uses T-scores normed to a European reference population (M = 50, SD = 10). Adjusted trajectories showed that children with low PA consistently scored approximately half a standard deviation below the normative mean, whereas those with high PA scored close to or within the normative range across all waves. The 4.31-point difference between high and low PA thus represents the difference between below-average and normatively expected well-being. For mental health problems, all PA groups scored within the normal range of the SDQ (≤13 points), indicating that PA-related differences reflect variation within normative functioning rather than shifts across clinical thresholds. From a population perspective, however, even within-range variation matters: the majority of the sample (56.6%) reported low PA levels, and moving a substantial proportion of these youth toward moderate activity levels could shift the population distribution of well-being meaningfully toward the normative mean.

Post-hoc comparisons between the high and medium PA groups indicate that this dose–response gradient was characterized by diminishing returns: the incremental differences between high and medium PA (HrQoL: *d* = 0.16; SDQ: *d* = −0.08) were approximately half the size of the corresponding medium-over-low contrasts (HrQoL: *d* = 0.22; SDQ: *d* = −0.12). This pattern aligns with findings from Chekroud et al.'s (55) analysis of 1.2 million US adults, which showed that exercising 3–5 times per week was associated with the lowest mental health burden, with no additional benefit at higher frequencies. Our findings extend this observation to children and adolescents under pandemic conditions, where access to organized sports and leisure activities was intermittently restricted. Together, these findings suggest that the transition from inactivity to moderate engagement may represent a qualitative shift in daily functioning—introducing structured routines, opportunities for social interaction, and experiences of competence—whereas additional activity beyond this threshold adds incrementally to these mechanisms without creating comparable new pathways. This interpretation is consistent with Lubans et al.'s (29) framework, in which psychosocial mediators such as social connectedness and self-efficacy may respond most strongly to the initial uptake of regular activity. Notably, the diminishing returns pattern was more pronounced for mental health problems than for HrQoL, suggesting that well-being continues to increase with additional PA, while reductions in mental health problems level off once youth are already functioning in the normative range. From a public health perspective, these findings are encouraging, as moderate PA levels may represent a more achievable target for inactive youth than daily activity.

Meta-analytic evidence shows similar effect sizes: a systematic review of 14 studies found small, positive associations between PA and HrQoL in 3- to 18-year-olds (Hedges' *g* = 0.12–0.30), with no evidence for moderation by age, gender, or SES (56). Likewise, Kohake et al.'s (30) review of pandemic-era studies reported higher well-being among active youth in 12 of 13 studies, mirroring pre-pandemic evidence of PA's psychological benefits (21, 29, 57). Taken together, our findings are consistent with a growing body of evidence that PA represents a small but reliable factor associated with higher well-being and less mental health problems in youth under crisis conditions (30).

The subscale analyses further differentiated these findings: PA was most strongly associated with fewer emotional symptoms and peer problems—both internalizing domains—and least with conduct problems, an externalizing domain. While PA group differences were significant across all four subscales, effects were consistently larger for internalizing domains (*d* = −0.10 to −0.19) than for externalizing domains (*d* = −0.07 to −0.11), suggesting that PA may be more connected to internalizing than to externalizing symptoms in youth. This gradient is consistent with longitudinal evidence showing that PA most reliably predicts reductions in emotional and peer problems, with weaker and less consistent associations for externalizing domains (58, 59). The age-dependent attenuation of the PA–peer problems association may reflect that structured physical activities serve as a particularly important context for social integration in childhood, whereas peer relationships in adolescence become increasingly independent of organized activity settings (29).

### 4.3 Bidirectionality and mechanisms

Although PA is well established as a modifiable factor associated with better mental health through neurobiological, behavioral, and psychosocial pathways (22, 23, 29), the relationship is likely bidirectional. Longitudinal data from the German MoMo cohort indicate that higher pre-pandemic HrQoL predicted greater subsequent PA, particularly among younger children and females, indicating that strong psychosocial resources (e.g., cognitive, motivational, social, and material support) facilitate active lifestyles during crises (18). Similarly, findings from the UK Millennium Cohort show that higher baseline well-being predicts greater subsequent PA, again highlighting the role of psychological and contextual resources in enabling sustained PA (60). Future research should examine bidirectional dynamics using dedicated panel designs such as random-intercept cross-lagged models.

Although the present study did not directly measure mediating pathways, the pattern of results invites speculative interpretation through the lens of Lubans et al.'s (29) conceptual model. The stronger associations between PA and HrQoL compared to mental health problems may suggest that psychosocial mechanisms—such as enhanced self-esteem, social connectedness, and a sense of competence—were particularly relevant in the present context. During the pandemic, when social interactions were severely curtailed, PA may have served as one of the few remaining avenues for social connection and structured daily routines, both of which are known to support well-being (24). The slightly stronger association of high PA with fewer mental health problems among older adolescents could reflect the greater role of self-regulatory mechanisms in this age group, whereby PA serves as a deliberate coping strategy for managing stress and negative affect—consistent with reports that adolescents commonly used PA as a coping strategy during the pandemic (61). Neurobiological pathways, including effects on endorphin release and sleep quality associated with regular PA (28, 29), may have further contributed. Future studies should incorporate measures of these proposed mediators to directly test which pathways are most operative during crisis conditions.

Taken together with our graded and stable associations, these considerations support the idea that prevention and intervention strategies should aim both to promote PA and to strengthen psychosocial resources that enable sustained engagement in PA.

### 4.4 Heterogeneity across subgroups and over time

In line with H2b, we tested whether the association between PA and mental health varied by age, gender, SES, or pandemic phase. The non-linear time effects in both models reflect pandemic phase-specific fluctuations in overall mental health, consistent with previous COPSY publications documenting the strongest impairments during lockdown periods and gradual recovery thereafter (6–8). However, most PA  × covariate interactions were non-significant after model reduction. In particular, PA–mental health associations remained stable across pandemic phases, as indicated by the non-significant PA × time interactions, suggesting that the relevance of PA for youth mental health was not confined to particular pandemic conditions but persisted as restrictions eased and organized activities resumed. Similarly, no significant moderation by gender or SES was found, indicating that once sufficiently active, children and adolescents showed comparable associations across demographic groups. One exception was a small but significant PA × age interaction emerged for mental health problems: the association of high PA was slightly stronger among older adolescents. Given that PA was commonly used as a coping strategy by adolescents during the COVID-19 pandemic (61), this may reflect age-related differences in how PA is experienced. Older youth may use PA more purposefully as a self-regulation strategy, thereby deriving greater psychological benefit, consistent with psychosocial mechanisms described by Lubans et al. (29). Overall, H2b was thus only partially supported, with evidence for moderation by age but not by gender, SES, or pandemic phase.

These findings stand in contrast to theoretical assumptions and population-level surveillance data suggesting that PA and mental health are closely linked to individual characteristics such as age, gender, and SES (13, 29, 35). For example, health monitoring data from the HBSC study show pronounced participation gaps in PA by age and gender. Only 10% of girls and 20% of boys meet WHO PA guidelines, with girls' activity declining most sharply over the last decade (19). Likewise, children and adolescents from lower-SES families report lower HrQoL and more mental health problems (9, 37, 38). They are also less likely to engage in organized sports and maintain long-term club involvement (36, 62, 63), and more often engage in informal activities such as walking or active play (64).

Despite well-documented socioeconomic and gender disparities in both PA participation and mental health outcomes, studies jointly analyzing PA, mental health, and socioeconomic factors remain rare (65). Our finding that PA-mental health associations did not differ significantly by SES or gender—with the exception of a small age interaction for mental health problems—suggests that the primary equity challenge may lie in access to PA opportunities rather than in differential benefit from PA itself. Descriptively, correlations between SES and both PA (*r* = .02) and mental health outcomes (*r* = .01–.08) were negligible in this sample (see Supplementary Material), consistent with the non-significant moderation findings. However, SES was operationalized solely through parental education (CASMIN classification), which captures only one dimension of socioeconomic position. A more comprehensive assessment including household income, neighbourhood deprivation, or access to recreational facilities might reveal stronger socioeconomic gradients in both PA participation and its mental health correlates. Future research should examine these intersections more systematically, investigating whether socioeconomic factors primarily constrain PA participation through structural barriers or additionally moderate the mental health benefits of PA among those who are active.

### 4.5 Strengths and limitations

Strengths of this study include (a) a large, population-based cohort with six repeated measures spanning critical pandemic and post-pandemic phases, (b) flexible mixed-effects models with random slopes and natural splines to capture individual and non-linear time trajectories, (c) comprehensive model diagnostics supporting model validity, and (d) use of psychometrically validated and widely used instruments for HrQoL and mental health.

Several limitations should also be noted. First, PA was assessed via a single self-report item, which cannot capture the duration, intensity, type, or context of PA and may be prone to recall and social desirability bias (16). Although this item has demonstrated acceptable validity in German youth samples (41, 42), self-report instruments generally overestimate PA levels compared to device-based measures such as accelerometry, and recent evidence suggests that accelerometer-measured PA may reveal stronger or different patterns of association with mental health outcomes (34). Nonetheless, brief self-report questionnaires remain the most practical approach for large population-based cohort studies conducted under pandemic constraints (16), and future research should integrate device-based PA measures to better characterize dose–response relationships and their underlying mechanisms. Moreover, the categorization into three discrete groups (low, medium, high), while following HBSC convention (19, 44) and enabling comparability across studies, involves a reduction of continuous variance that may attenuate dose-response estimates. The specific cut-points are convention-based and chosen primarily for interpretability. Alternative categorizations could yield different effect estimates. Our sensitivity analysis using a binary WHO indicator (≥60 min/day) yielded consistent results, providing some reassurance regarding robustness across different operationalizations.

Second, different perspectives (self-reported PA vs. parent-reported outcomes) may reduce common-method bias but can also attenuate associations, yielding conservative estimates. Third, residual confounding by unmeasured factors such as sleep, screen time, or living environment cannot be fully excluded. Fourth, this study was conducted in Germany, a high-income Western European country. Findings may not generalize to low- and middle-income countries or to contexts with different PA cultures and pandemic policy responses. Finally, the observational design precludes causal inference; quasi-experimental or intervention studies of PA promotion during crises would be valuable to strengthen causal claims.

### 4.6 Public health implications

The findings of this study suggest that engaging in sufficient PA during the COVID-19 pandemic was associated with better mental health and well-being among children and adolescents. Importantly, associations were somewhat stronger for HrQoL than for mental health problems, highlighting that PA may contribute more to enhancing well-being than to reducing pathology. This distinction supports a salutogenic, health-promotion perspective that emphasizes PA as a resource for strengthening well-being and positive functioning, rather than solely a preventive measure against mental illness.

Given the sustained global declines in youth PA over the past decade (13) and pandemic-related disruptions (18), addressing PA promotion remains a major public-health and health-promotion priority. In crisis contexts, when organized sports, schools, and community spaces are restricted, public-health authorities should maintain safe, low-barrier PA opportunities (e.g., safe outdoor spaces, access to school playgrounds). Moreover, effective interventions require action across individual, family and community levels: municipal “healthy community” initiatives can integrate active transport, public space design, and cross-sector partnerships between youth services and sports clubs to create environments that sustain youth PA. Such community-based interventions may positively influence PA and mental health and well-being through mechanisms like social cohesion (66).

Integrating PA into school-based mental health programs and family routines may be particularly promising. Importantly, our results indicate that already moderate PA levels were associated with meaningful benefits, suggesting that daily vigorous activity may not be necessary for such benefits to emerge. In schools, regular but feasible opportunities for movement may support well-being—particularly relevant given the established association of both PA and mental health with academic performance in youth (20, 67). In families, shared physical activities several times per week can foster bonding and strengthen familial resources associated with enhanced resilience during crises (68, 69).

### 4.7 Conclusions and future directions

Medium and high levels of PA were consistently associated with higher HrQoL and fewer mental health problems during and after the COVID-19 pandemic among German children and adolescents. While effects were small in size, their robustness across multiple outcomes and time points underscores the public health relevance of PA as a reliable resource for youth mental health and well-being amid global crises. The stronger associations for HrQoL than for mental health problems suggest that PA may be particularly effective in enhancing positive well-being through health-promotion rather than merely reducing symptoms.

Given its modifiable nature, low cost, and wide-ranging benefits, PA should be prioritized in both crisis preparedness and long-term youth mental health strategies. Future research should (1) integrate device-based PA measures, (2) test causal pathways in (quasi-) experimental or intervention designs, (3) examine bidirectional dynamics between PA and both mental health and well-being using panel designs such as random-intercept cross-lagged models, and (4) identity social, contextual and environmental factors that facilitate the development and implementation of strategies to help children and adolescents increase their PA levels. Policymakers and practitioners are encouraged to invest in evidence-based, context-sensitive PA programs and environments that can support against the mental health impacts of future crises and support sustained psychosocial development in youth.

## Data Availability

The raw data supporting the conclusions of this article will be made available by the authors, without undue reservation.
