# Supplementary figures and images for: Physical activity and mental health in children and adolescents during and after the COVID-19 pandemic: findings from the six-wave German COPSY study
Source: Front Sports Act Living. 2026 Apr 8;8:1776510. doi: 10.3389/fspor.2026.1776510 (PMC13099543; doi:10.3389/fspor.2026.1776510)

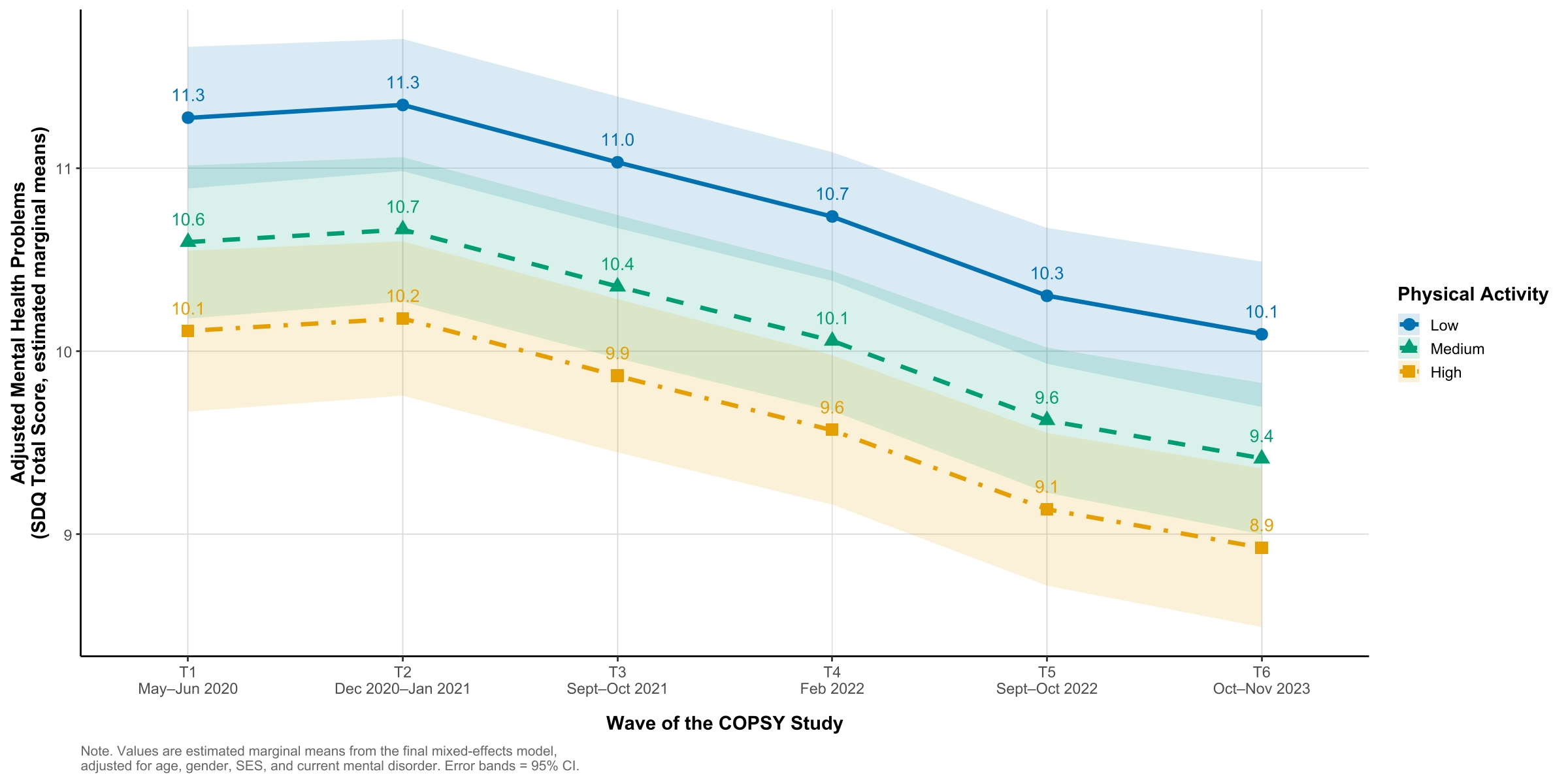

Supplement: Supplementary file 1 [file Image1.jpeg]

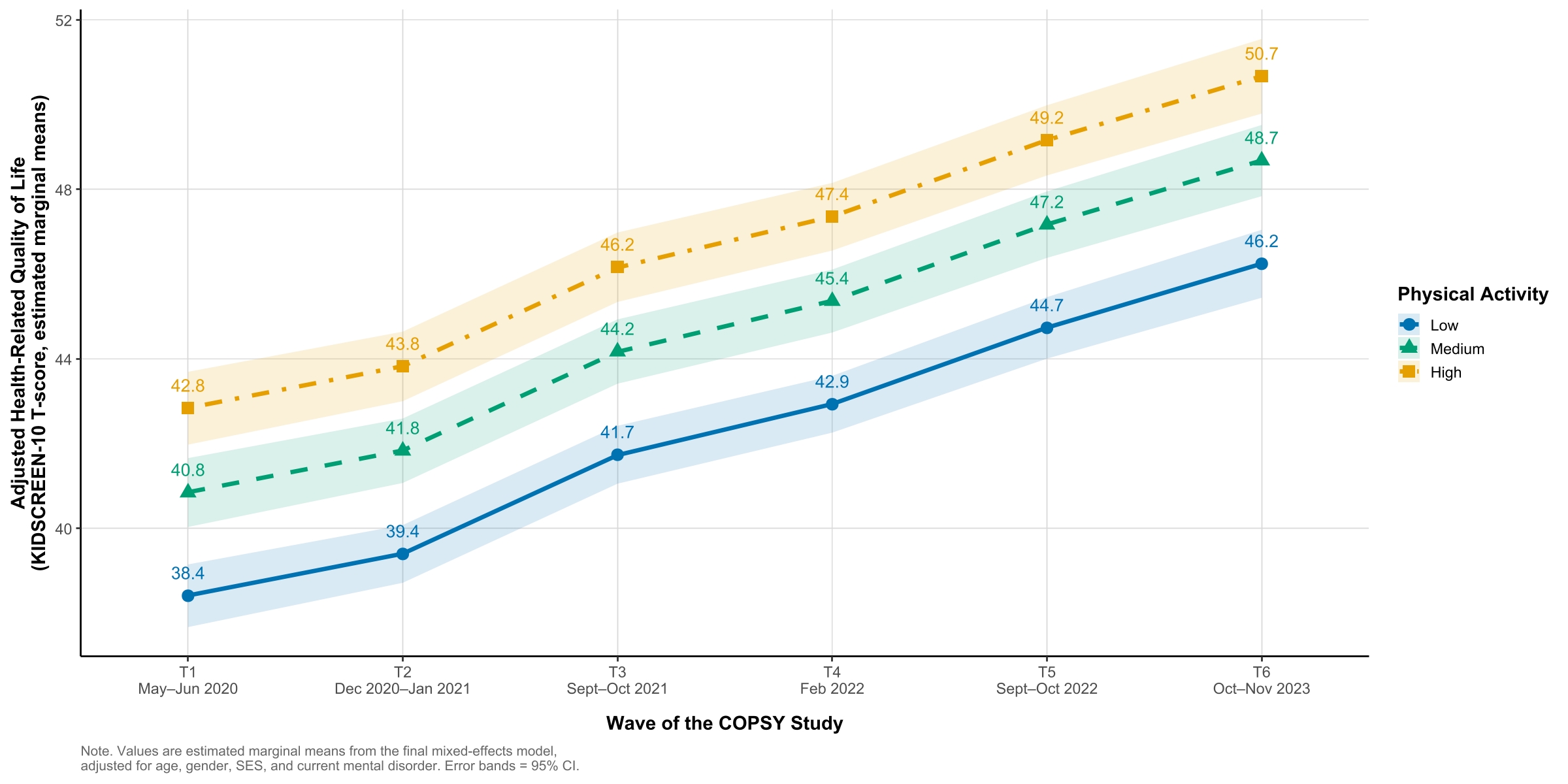

Supplement: Supplementary file 2 [file Image2.jpeg]
