# Supplementary material for: Physical activity and mental health in children and adolescents during and after the COVID-19 pandemic: findings from the six-wave German COPSY study
Source: Front Sports Act Living. 2026 Apr 8;8:1776510. doi: 10.3389/fspor.2026.1776510 (PMC13099543; doi:10.3389/fspor.2026.1776510)
Supplement: Supplementary file 3 [file Table1.docx]

***Supplement Material***

*Supplementary Table 1*. Intercorrelations between key study variables

| **Variable** | **1** | **2** | **3** | **4** | **5** | **6** | **7** |
| --- | --- | --- | --- | --- | --- | --- | --- |
| 1. HrQoL | — |  |  |  |  |  |  |
| 2. Mental health problems | −.65*** | — |  |  |  |  |  |
| 3. Physical activity | .24*** | −.16*** | — |  |  |  |  |
| 4. Age | .02 | −.16*** | −.08*** | — |  |  |  |
| 5. Sex | .00 | −.05*** | −.07*** | .03* | — |  |  |
| 6. Socioeconomic status | .01 | −.08*** | .02 | .05*** | −.07*** | — |  |
| 7. Current mental disorder | −.18*** | .32*** | −.08*** | .00 | −.02 | −.04*** | — |
| 8. Time | .26*** | −.07*** | .11*** | .00 | .00 | .04*** | .03* |

Note. Values are r (Pearson correlation coefficients). HrQoL = health-related quality of life. * *p* < .05, *** *p* < .001. All correlations computed with pairwise deletion.

*Supplementary Table 2.* Results of the final linear mixed model for health-related quality of life in children and adolescents

| **Predictor** | ***b*** | **Wald  95% - *CI*** | **Bootstrap 95% - *CI*** |  | ***SE*** | ***t*** | ***p*-value** |
| --- | --- | --- | --- | --- | --- | --- | --- |
| Intercept | 40.18 | [39.25; 41.12] | [39.46; 40.66] |  | 0.48 | 84.05 | < .001 |
| *Low PA (Ref.)* |  |  |  |  |  |  |  |
| Medium PA | 2.51 | [2.00; 3.02] | [2.33; 3.24] |  | 0.26 | 9.60 | < .001 |
| High PA | 4.31 | [3.70; 4.92] | [4.19; 5.23] |  | 0.31 | 13.79 | < .001 |
| *Time* |  |  |  |  |  |  |  |
| Spline component 1 | 5.83 | [5.05; 6.62] | [5.28; 6.68] |  | 0.40 | 14.60 | < .001 |
| Spline component 2 | 8.87 | [7.78; 9.99] | [7.79; 9.75] |  | 0.56 | 15.82 | < .001 |
| Spline component 3 | 6.87 | [6.25; 7.49] | [6.46; 7.46] |  | 0.32 | 21.76 | < .001 |
| Age (centered) | 0.27 | [0.09; 0.44] | [0.17; 0.38] |  | 0.09 | 2.93 | .003 |
| *Male (Ref.)* |  |  |  |  |  |  |  |
| Female | 0.33 | [–0.51; 1.16] | [0.01; 0.68] |  | 0.42 | 0.77 | .443 |
| *High SES (Ref.)* |  |  |  |  |  |  |  |
| Low SES | 0.51 | [–0.56; 1.59] | [0.34; 1.66] |  | 0.55 | 0.93 | .351 |
| Medium SES | –0.32 | [–1.12; 0.47] | [–0.80; 0.29] |  | 0.40 | –0.79 | .427 |
| *No current mental disorder (Ref.)* |  |  |  |  |  |  |  |
| Current mental disorder | –4.32 | [–5.31; –3.33] | [–6.53; –5.01] |  | 0.50 | –8.59 | < .001 |
| Medium PA × Age | –0.12 | [–0.33; 0.08] | [–0.29; 0.06] |  | 0.10 | –1.20 | .232 |
| High PA × Age | 0.20 | [–0.04; 0.43] | [0.00; 0.38] |  | 0.12 | 1.65 | .099 |

*Note.* All regression coefficients are unstandardized. PA = Physical Activity. SES = socioeconomic status. Time modeled as natural cubic spline (*df* = 3). Age is grand-mean centered. Model fit: marginal *R*² = 0.11; conditional *R²* = 0.65. *N* = 6,412 observations from 1,807 adolescents

*Supplementary Table 3.* Results of the final linear mixed model for mental health problems in children and adolescents

| **Predictor** | **b** | **Wald  95% - CI** | **Bootstrap  95% - CI** |  | **SE** | **t** | **p-value** |
| --- | --- | --- | --- | --- | --- | --- | --- |
| Intercept | 9.69 | [9.18; 10.20] | [9.30; 9.93] |  | 0.26 | 37.60 | < .001 |
| *Low PA (Ref.)* |  |  |  |  |  |  |  |
| Medium PA | –0.73 | [–0.98; –0.49] | [–1.17; –0.72] |  | 0.12 | –5.90 | < .001 |
| High PA | –1.23 | [–1.53; –0.94] | [–1.69; –1.16] |  | 0.15 | –8.23 | < .001 |
| *Time* |  |  |  |  |  |  |  |
| Spline component 1 | –1.14 | [–1.51; –0.78] | [–1.54; –0.83] |  | 0.19 | –6.13 | < .001 |
| Spline component 2 | –0.98 | [–1.49; –0.47] | [–1.42; –0.45] |  | 0.26 | –3.75 | < .001 |
| Spline component 3 | –1.28 | [–1.56; –1.00] | [–1.58; –1.09] |  | 0.14 | –8.90 | < .001 |
| Age (centered) | –0.41 | [–0.50; –0.31] | [–0.48; –0.38] |  | 0.05 | –8.24 | < .001 |
| *Male (Ref.)* |  |  |  |  |  |  |  |
| Female | –0.57 | [–1.05; –0.09] | [–0.76; –0.42] |  | 0.24 | –2.34 | .019 |
| *High SES (Ref.)* |  |  |  |  |  |  |  |
| Low SES | 0.67 | [0.10; 1.24] | [0.45; 1.20] |  | 0.29 | 2.32 | .021 |
| Medium SES | 0.37 | [–0.04; 0.78] | [0.13; 0.71] |  | 0.21 | 1.75 | .080 |
| *No current mental disorder (Ref.)* |  |  |  |  |  |  |  |
| Current mental disorder | 3.52 | [3.04; 4.00] | [4.23; 5.06] |  | 0.25 | 14.31 | < .001 |
| Medium PA × Age | 0.09 | [0.00; 0.19] | [0.06; 0.21] |  | 0.05 | 1.90 | .058 |
| High PA × Age | 0.11 | [0.00; 0.23] | [0.03; 0.22] |  | 0.06 | 1.97 | .049 |

*Note.* All regression coefficients are unstandardized. PA = Physical Activity. SES = socioeconomic status. Time modeled as natural cubic spline (*df* = 3). Age is grand-mean centered. Model fit: marginal *R*² = 0.06; conditional *R²* = 0.75. *N* = 6,412 observations from 1,807 adolescents.

*Supplementary Table 4.* Results of the final linear mixed model for the SDQ subscales in children and adolescents

|  | **Emotional symptoms** | | **Conduct problems** | | **Hyperactivity** | | **Peer problems** | |
| --- | --- | --- | --- | --- | --- | --- | --- | --- |
| **Coefficient** | ***b* [95% CI]** | ***p*** | ***b* [95% CI]** | ***p*** | ***b* [95% CI]** | ***p*** | ***b* [95% CI]** | ***p*** |
| Intercept | 1.55 [1.36, 1.74] | <.001 | 1.98 [1.83, 2.13] | <.001 | 3.76 [3.57, 3.94] | <.001 | 2.39 [2.22, 2.55] | <.001 |
| *Low PA (Ref.)* |  |  |  |  |  |  |  |  |
| Medium PA | −0.23 [−0.33, −0.13] | <.001 | −0.15 [−0.23, −0.08] | <.001 | −0.18 [−0.27, −0.09] | <.001 | −0.27 [−0.35, −0.18] | <.001 |
| High PA | −0.40 [−0.52, −0.28] | <.001 | −0.19 [−0.29, −0.10] | <.001 | −0.36 [−0.47, −0.25] | <.001 | −0.36 [−0.47, −0.26] | <.001 |
| *Time* |  |  |  |  |  |  |  |  |
| Spline component 1 | −0.04 [−0.19, 0.12] | .629 | −0.29 [−0.41, −0.18] | <.001 | −0.52 [−0.66, −0.38] | <.001 | −0.30 [−0.43, −0.17] | <.001 |
| Spline component 2 | 0.55 [0.33, 0.76] | <.001 | −0.21 [−0.37, −0.04] | .015 | −1.04 [−1.24, −0.85] | <.001 | −0.27 [−0.45, −0.08] | .005 |
| Spline component 3 | −0.09 [−0.21, 0.03] | .134 | −0.31 [−0.40, −0.22] | <.001 | −0.67 [−0.77, −0.56] | <.001 | −0.24 [−0.34, −0.13] | <.001 |
| Age (centered) | −0.08 [−0.11, −0.04] | <.001 | −0.08 [−0.11, −0.05] | <.001 | −0.22 [−0.25, −0.18] | <.001 | −0.04 [−0.07, −0.01] | .008 |
| *Male (Ref.)* |  |  |  |  |  |  |  |  |
| Female | 0.49 [0.32, 0.66] | <.001 | −0.17 [−0.30, −0.04] | .011 | −0.66 [−0.83, −0.49] | <.001 | −0.23 [−0.38, −0.09] | .002 |
| *High SES (Ref.)* |  |  |  |  |  |  |  |  |
| Low SES | 0.00 [−0.22, 0.22] | .998 | 0.30 [0.13, 0.47] | <.001 | 0.28 [0.07, 0.49] | .009 | 0.15 [−0.04, 0.34] | .120 |
| Medium SES | −0.00 [−0.16, 0.16] | .975 | 0.11 [−0.01, 0.24] | .070 | 0.13 [−0.03, 0.28] | .102 | 0.15 [0.01, 0.29] | .033 |
| *No current mental disorder (Ref.)* |  |  |  |  |  |  |  |  |
| Current mental disorder | 1.50 [1.31, 1.70] | <.001 | 0.62 [0.47, 0.77] | <.001 | 1.01 [0.83, 1.19] | <.001 | 0.78 [0.61, 0.95] | <.001 |
| Medium PA × Age | 0.02 [−0.02, 0.06] | .349 | 0.02 [−0.01, 0.05] | .264 | 0.03 [−0.00, 0.07] | .079 | 0.04 [0.01, 0.07] | .025 |
| High PA × Age | 0.01 [−0.04, 0.06] | .719 | 0.03 [−0.00, 0.07] | .084 | 0.03 [−0.02, 0.07] | .260 | 0.05 [0.01, 0.09] | .010 |

*Note.* N = 6,412 person–wave observations from 1,807 participants. All models include random intercepts and random slopes for time. PA = physical activity; SDQ = Strengths and Difficulties Questionnaire. Reference categories: PA = low (0–2 days/week); SES = high (parental education); Gender = male. Time modelled using natural cubic splines (ns, df = 3).
